# Sarcopenia beyond muscle: endocrine–metabolic regulation and inter-tissue crosstalk across the muscle–tendon unit

**DOI:** 10.3389/fendo.2026.1935289

**Published:** 2026-09-25

**Authors:** Guang Ta, Li Li, Cai Jing, Xinxu Chen, Seung-Jun Jeong, Dongryeol Ryu, Shibo Wei

**Affiliations:** 1 Affiliated Hospital of Changchun University of Chinese Medicine, Changchun, China; 2 School of Basic Medicine, Faculty of Medicine, Dalian University of Technology, Dalian, China; 3 Department of Biomedical Science and Engineering, Gwangju Institute of Science and Technology (GIST), Gwangju, Republic of Korea

**Keywords:** extracellular matrix remodeling, muscle aging, muscle-tendon crosstalk, myotendinous junction, sarcopenia, tendon aging

## Abstract

Sarcopenia is characterized by a progressive loss of muscle strength and physical performance that frequently exceeds the decline predicted by muscle mass alone. This discrepancy indicates that functional deterioration is shaped not only by the force-generating capacity of skeletal muscle, but also by the tissues responsible for transmitting and integrating that force. In this Review, we examine sarcopenia through the biology of the muscle-tendon unit, encompassing skeletal muscle, tendon, and their specialized interface. We discuss how systemic aging processes, including metabolic dysfunction, inflammaging, and vascular insufficiency, produce tissue-specific and potentially asynchronous effects on muscle contractile capacity, tendon matrix remodeling, and myotendinous junction (MTJ) integrity. Much of the evidence for tendon and MTJ remodeling derives from physiological aging and experimental models, whereas their characteristic phenotypes in clinically defined sarcopenia remain insufficiently characterized. Since muscle, the MTJ, and tendon form a serial force-transmission pathway, differential remodeling across these components may create a mismatch between force-generating and force-transmitting capacities. We further evaluate the strength of evidence for inter-tissue communication, distinguishing experimentally supported muscle-to-tendon regulation from emerging tendon-to-muscle effects demonstrated primarily in co-culture, conditioned-medium, and engineered-interface systems. Particular emphasis is placed on the myotendinous junction as a structurally specialized interface where mechanical forces, extracellular matrix organization, and local signaling converge. Age-related disruption of this interface may reduce force-transfer efficiency and magnify the functional consequences of muscle weakness. Finally, we consider how the muscle–tendon unit framework may refine exercise prescription, functional phenotyping, and the evaluation of future tissue-specific interventions while remaining complementary to established diagnostic criteria and current exercise-based management. Together, these observations identify impaired coordination across the muscle–tendon unit as a potentially important contributor to loss of strength and physical performance in sarcopenia.

## 1 Introduction

Sarcopenia is conceptualized as a multifactorial disorder of skeletal muscle integrity in which diminished muscle strength, rather than loss of muscle mass alone, constitutes the principal diagnostic feature (1, 2). Reduced muscle quantity and compromised muscle quality underpin the diagnosis, whereas impaired physical performance reflects a more advanced stage of functional decline (3, 4). This conceptual evolution has shifted the field beyond mass-centric definitions toward an integrated view in which age-related deterioration arises from alterations in muscle composition, microarchitectural integrity, regenerative capacity, and neuromuscular coordination, rather than from muscle atrophy alone (5, 6). This broader framework is increasingly supported by convergent evidence from human studies and experimental models. Declining mobility and force in older adults cannot be adequately explained by loss of muscle mass alone, but instead arise from coordinated changes in the skeletal muscle microenvironment, including extracellular matrix (ECM) remodeling, fibrosis, defective mechanotransductive signaling and progressive disruption of neuromuscular connectivity (7). Emerging human-centered analyses further suggest that connective-tissue remodeling and alterations in interfacial mechanics make substantive contributions to sarcopenic dysfunction that are insufficiently captured by conventional measurements of muscle size alone (8). Current consensus frameworks, including EWGSOP2 and AWGS 2019, remain centered on measurements of muscle strength, muscle quantity or quality, and physical performance. The muscle–tendon unit perspective considered here is therefore intended as a complementary mechanistic framework for understanding functional heterogeneity, rather than as a replacement for these established diagnostic criteria.

Within this expanded pathophysiological landscape, tendon warrants greater attention. Tendon is not merely a passive cable interposed between muscle and bone, but a mechanically responsive and biologically active connective tissue whose material properties are integral to force transmission, elastic energy storage, movement efficiency and rate of torque development (9, 10). Aging is closely associated with reduced tendon stiffness and elastic modulus in major lower-limb tendons, which occur in parallel with declines in muscle strength and architectural remodeling. Increased tendon compliance in older adults may impair rapid force transmission and thereby amplify deficits in balance recovery, gait performance and explosive force production. Importantly, these properties remain at least partly trainable, indicating that tendon aging is functionally significant but not biologically fixed (11, 12).

The biological relationship between muscle and tendon, however, is best viewed not as a single phenomenon but as a continuum of interaction. Muscle contraction and tendon loading are obligatorily coupled by mechanics. Meanwhile, both tissues are shaped by shared systemic drivers of aging, including inflammation, metabolic dysfunction, vascular insufficiency and physical inactivity. Additionally, accumulating evidence points to local signal-mediated communication between the two tissues and across their interface (13). In this Review, we distinguish these forms of interaction explicitly: mechanical coupling refers to force-dependent interdependence between muscle and tendon; shared systemic influences describe parallel exposure of both tissues to common endocrine, metabolic, inflammatory, or vascular conditions; and crosstalk is used more specifically to denote inter-tissue communication mediated by biochemical signals, extracellular vesicles, matrix-associated cues, or other locally transmitted regulatory information. These layers should not be regarded as competing explanations, but as biologically nested modes of interaction through which aging perturbs the muscle-tendon unit. In this circumstance, the relevant question is not whether muscle and tendon are related, which are inseparably linked in both structure and function, but how mechanical interdependence, shared pathobiology and direct signaling are integrated across different physiological and pathological contexts. Adult biochemical crosstalk remains less completely defined than developmental or biomechanical coupling (14). Current evidence is directionally asymmetric: muscle-to-tendon signaling is supported by developmental and experimentally decoupled models, whereas tendon-to-muscle regulation is derived primarily from co-culture, conditioned-medium, and engineered-interface systems, with direct *in vivo* validation still lacking. This reappraisal is further justified by the recognition that skeletal muscle is a secretory organ capable of releasing myokines, growth factors and extracellular vesicles with local as well as systemic effects (15). In parallel, tendon and the ECM at the muscle-tendon interface are increasingly understood to be biologically instructive rather than structurally inert, with emerging evidence that they regulate cell behavior, mechanosensing and regenerative responses (16, 17). These observations support the investigation of multiple modes of muscle–tendon interaction, while indicating that adult bidirectional biochemical crosstalk remains an emerging and incompletely validated component of muscle–tendon unit biology.

Importantly, the temporal relationship between muscle decline and tendon aging is likely to vary across biological and clinical contexts. Tendon aging may occur independently as part of intrinsic tissue aging, develop secondarily to reduced mechanical loading associated with muscle weakness, or coexist with muscle deterioration under shared systemic influences. These trajectories are not mutually exclusive, and their relative contributions may differ among individuals and across stages of sarcopenia. Alterations in tendon and myotendinous junction properties may consequently influence how reductions in muscle capacity are translated into losses of strength and physical performance, regardless of whether they arise before, after, or in parallel with muscle deterioration (18, 19). Considering muscle, tendon, and their interface together therefore provides a mechanistic basis for understanding why functional decline in sarcopenia may exceed that predicted by muscle mass loss alone (20). Notably, the current evidence base must be interpreted according to its biological context. Whereas muscle abnormalities are well documented in clinically defined sarcopenia, much of the evidence concerning tendon remodeling, MTJ adaptation, and inter-tissue regulation comes from studies of physiological aging, altered mechanical loading, developmental models, or reductionist experimental systems. Accordingly, the muscle–tendon unit framework developed here represents a mechanistic synthesis and a set of testable hypotheses for sarcopenia rather than evidence for an already established sarcopenia-specific tendon or MTJ phenotype.

Particular attention should therefore be directed to the myotendinous junction (MTJ), the specialized interface at which mechanical stress, ECM specialization and intercellular signaling converge (21). The MTJ is distinguished by a highly folded architecture and a molecular composition that is not simply intermediate between muscle and tendon, but partially unique to the interface (22). Developmental, proteomic and mechanobiological studies increasingly suggest that this region functions as a regulatory niche relevant to tissue alignment, force transmission, adaptation and repair (23, 24). Age-related remodeling of the MTJ may therefore alter how changes in muscle and tendon are integrated and translated into force transmission, making the interface a potentially important contributor to functional decline in sarcopenia.

In this review, we examine muscle–tendon interactions in sarcopenia within a framework that distinguishes mechanical coupling, shared aging biology, and direct inter-tissue signaling across the muscle–tendon unit. We outline systemic drivers that concurrently remodel muscle and tendon, then evaluate evidence for muscle-to-tendon and tendon-to-muscle biochemical communication, and consider the myotendinous junction as an interface at which these processes are integrated. By examining sarcopenia at the level of the muscle–tendon unit, we consider how alterations in force generation, force transmission and interface function may collectively shape the severity and heterogeneity of functional decline and influence the identification of appropriate therapeutic targets.

To improve transparency in evidence selection, this Review was developed through targeted searches of PubMed and Web of Science together with backward reference tracing of relevant primary and review literature. Evidence was selected to represent the principal biological and biomechanical dimensions of the muscle–tendon unit, including sarcopenia, muscle and tendon aging, MTJ remodeling, ECM biology, force transmission, inter-tissue signaling, and exercise adaptation. Human data were prioritized whenever available, whereas animal, developmental, unloading, and reductionist experimental models were included primarily to address mechanistic questions that remain unresolved in clinically defined sarcopenia. Since these evidence sources differ substantially in biological scale and translational relevance, they were interpreted hierarchically rather than equivalently, with greater weight given to human and clinically characterized data and experimental findings used to establish mechanistic plausibility and generate testable hypotheses.

## 2 Shared systemic drivers and their unit-specific functional consequences

### 2.1 Metabolic and mitochondrial dysregulation

Mitochondrial dysfunction represents one of the most robustly supported hallmarks of skeletal muscle aging. In aged muscle, impaired mitochondrial biogenesis, defective mitophagy, redox imbalance and energetic insufficiency converge to limit ATP availability, compromise contractile performance and promote anabolic resistance (25, 26). Importantly, these alterations do not merely reflect an energetically depleted state but rather a coordinated reprogramming toward a degenerative phenotype (**Figure 1**). Age-dependent upregulation of miR-29 suppresses IGF-1, p85α and B-myb, thereby impairing muscle progenitor proliferation and promoting senescence-like features (27). In parallel, decline of the NAD^+^-SIRT1/3 signaling axis disrupts mitochondrial protein deacetylation and antioxidant defenses, leading to accumulation of oxidative damage and impaired mitochondrial turnover (28, 29). Concomitantly, attenuation of AMPK-PGC1α activity reduces mitochondrial biogenic capacity and shifts muscle toward a glycolytic, fatigue-prone phenotype (30).

**Figure 1:**
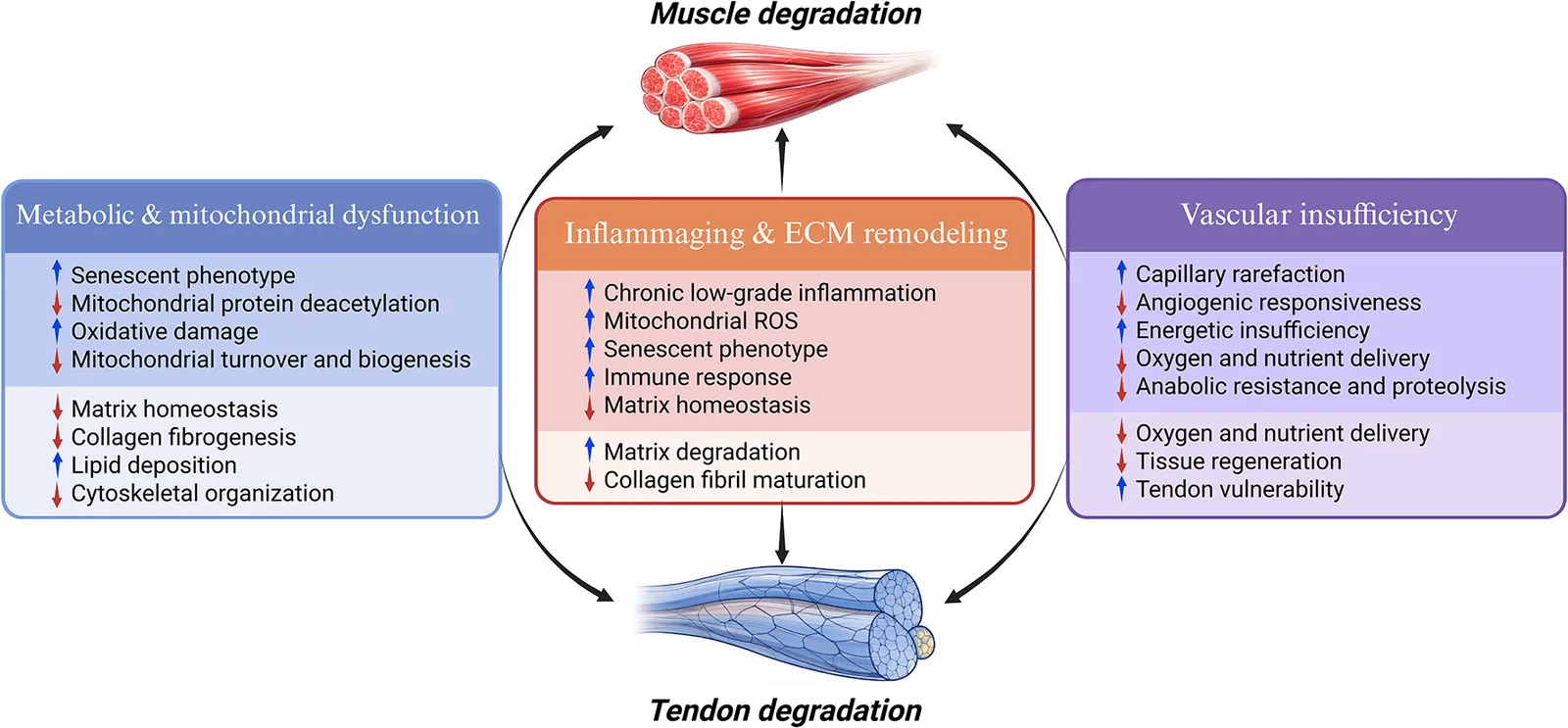
Shared systemic drivers and their differential consequences across muscle–tendon unit. Overview of metabolic and mitochondrial dysfunction, inflammaging with ECM remodeling, and vascular insufficiency. These processes are not specific to muscle and tendon but can produce tissue-dependent effects on muscle contractile capacity, intramuscular ECM organization, tendon matrix maintenance, and MTJ integrity. Their convergence may generate asynchronous remodeling and a mismatch between force generation and force transmission. Arrows indicate mechanistic associations and convergence rather than an established bidirectional signaling pathway between muscle and tendon. ECM, extracellular matrix; MTJ, myotendinous junction.

Although comparatively less developed, the available evidence on tendon points toward a broadly convergent direction, although it derives largely from studies of aging rather than clinically defined sarcopenia. Aging tendon is increasingly characterized by reduced cellular fitness, impaired matrix maintenance and heightened stress susceptibility (31). In human Achilles tendon progenitor cells, aging is associated with reduced self-renewal and clonogenicity, premature senescence with increased p16, delayed migration and wound closure, and marked alterations in actin dynamics and cell-matrix interactions (32). Similarly, in mouse Achilles tendon, age-related decline in SPARC is linked to defective collagen fibrogenesis, reduced force tolerance, altered cytoskeletal organization and increased lipid deposition (33), indicating that metabolic stress, matrix failure and loss of structural competence are tightly intertwined in tendon aging. These features are increasingly interpreted within a broader framework of metabolic insufficiency and impaired cellular energy homeostasis in aging tendon, aligning conceptually with the mitochondrial and redox dysregulation observed in skeletal muscle.

These observations support a model in which both muscle and tendon aging occur within a common catabolic milieu characterized by oxidative stress, impaired proteostasis and diminished reparative capacity. What is not yet established is that mitochondrial or metabolic failure in one compartment directly imposes the same failure on the other through a defined *in vivo* signaling axis. At present, the strongest interpretation is therefore one of shared systemic vulnerability with tissue-specific consequences. In muscle, energetic failure directly limits contraction and adaptive protein synthesis, whereas in tendon it may constrain the slower processes of matrix maintenance and repair. Because these tissues occupy sequential positions in the same force-transmission pathway, unequal rates of metabolic decline may create a mismatch between force generation and the capacity of tendon and the MTJ to transmit and adapt to that force.

### 2.2 Inflammaging disrupts ECM-dependent force transmission

Chronic low-grade inflammation is a defining feature of musculoskeletal aging and is increasingly inseparable from ECM remodeling. In skeletal muscle, inflammaging is sustained by mitochondrial ROS, senescence-associated secretory activity, altered immune signaling and redox-sensitive inflammatory pathways, collectively shifting remodeling away from adaptation and towards fibrosis, proteolysis and regenerative failure (34) (**Figure 1**). Human skeletal muscle aging reviews now place inflammation and ECM dysfunction among the major determinants of declining muscle quality and function (35). This ECM dimension is significant since muscle force is not transmitted solely through the contractile axis of myofiber; instead, a substantial proportion is conveyed laterally through connective-tissue networks, indicating that matrix fibrosis, altered collagen organization and aberrant stiffness are not secondary curiosities but primary determinants of muscle function (35). Age-related remodeling of intramuscular connective tissue may therefore help explain why losses of muscle strength can exceed those predicted by muscle mass alone. In the context of sarcopenia, impaired matrix-mediated force transmission may plausibly contribute to functional decline, alongside alterations in muscle quantity, quality, innervation, and intrinsic contractile capacity (36).

Tendon aging shows a closely related pattern, although its biology is expressed through a different tissue architecture. One informative study demonstrated that aging tenocytes exhibit increased MMP-2 and MMP-9 activity, changes that provide a plausible mechanism by which aging shifts tendon homeostasis towards matrix degradation and mechanically inferior tissue (37). More broadly, age-related loss of SPARC compromises collagen fibril maturation and tissue strength, whereas aged tendon stem/progenitor cells display cell-matrix dysregulation (32, 33). These findings imply that tendon inflammaging is inseparable from progressive loss of matrix competence during aging, although direct evidence linking this process to a distinct tendon phenotype in clinically defined sarcopenia remains limited.

The relevance of inflammaging to the muscle–tendon unit therefore lies not in its uniqueness as an aging process, but in the mechanically coupled consequences of tissue-specific ECM remodeling. In muscle, fibrosis and disorganization of the endo- and perimysial networks can impair lateral force transmission from myofibers, whereas in tendon, increased matrix degradation, defective collagen maturation, and altered fibrillar organization can modify stiffness and longitudinal force transfer. When these changes occur concurrently or at different rates, contractile force may be generated within a muscle whose intramuscular matrix, tendon, and MTJ no longer transmit that force efficiently (14). This sequential disruption of force transfer provides a unit-specific mechanism through which a generalized inflammatory environment may contribute to sarcopenic weakness. However, it remains unresolved whether inflammation originating in one compartment directly propagates a degenerative program to the other *in vivo*.

### 2.3 Vascular insufficiency as a systemic and asymmetric constraint

Reduced vascular perfusion is increasingly recognized as an integral component of sarcopenia (38). In skeletal muscle, disruption of vascular homeostasis, including capillary rarefaction and impaired angiogenic responsiveness, limits oxygen and nutrient delivery, restricts hormonal access and waste clearance, and thereby exacerbates mitochondrial dysfunction, anabolic resistance and proteolysis (39, 40) (**Figure 1**). Human studies provide convergent support for this framework. Aging is associated with reduced capillary density and blunted microvascular responsiveness to both insulin and exercise, changes that constrain perfusion-mediated substrate delivery and metabolic flexibility (41). These vascular deficits are closely linked to impaired glucose uptake, reduced oxidative capacity and declining muscle performance (42). Moreover, imaging and physiological analyses indicate that perfusion is spatially heterogeneous within skeletal muscle, with deficits preferentially affecting metabolically active regions, thereby amplifying regional energetic insufficiency (43).

For tendon, the evidentiary landscape remains comparatively less developed, yet supports a broadly consistent interpretation. Relative to skeletal muscle, tendon is intrinsically less vascularized, and its maintenance and repair operate under constrained trophic conditions. Age-related deterioration of tendon stem/progenitor cell function, together with impaired matrix synthesis and reduced adaptive capacity, is therefore likely to be further constrained within a vascular environment characterized by limited oxygen delivery, restricted nutrient availability and diminished regenerative support (44). Current evidence thus positions vascular insufficiency in tendon primarily as a determinant of tissue vulnerability (45), although its role as a fully delineated aging pathway remains incompletely resolved and has not been specifically defined in clinically characterized sarcopenia.

The significance of vascular insufficiency within the muscle–tendon unit is therefore asymmetric rather than uniform. Skeletal muscle requires rapid, activity-dependent perfusion to sustain contraction, substrate uptake, and training adaptation, whereas tendon operates under lower baseline vascularity and depends on slower trophic support for matrix turnover and repair. A common decline in vascular support may consequently produce immediate energetic and anabolic limitations in muscle but more gradual loss of matrix maintenance and adaptive capacity in tendon. Since muscle force and tendon loading are obligatorily coupled, these different temporal responses may progressively uncouple improvements or declines in force generation from the capacity for force transmission. Therefore, vascular insufficiency should be viewed as a systemic constraint that can amplify muscle–tendon mismatch, rather than as a muscle–tendon-specific disease mechanism in itself. Whether vascular decline directly synchronizes degeneration across the two tissues through a defined signaling pathway remains unresolved.

### 2.4 Convergence of systemic aging on force generation-transmission mismatch

One of the most compelling reasons to consider sarcopenia at the level of muscle-tendon unit comes from human biomechanics. Across studies of older adults, reductions in triceps surae and quadriceps strength are accompanied by declines in tendon stiffness and biomechanical properties, whereas changes in tendon cross-sectional area are less consistent (11). This pattern suggests that aging alters not only muscle performance, but also the material behavior of the tendon through which force must be transmitted (12). The consequence is not merely weaker tissue, but slower and less efficient transfer of force, especially during rapid or reactive tasks.

The concurrent presence of muscle weakness and altered tendon properties does not, however, establish a tendon-first sequence. Human unloading experiments demonstrate that tendon mechanical properties are strongly dependent on habitual muscle-generated loading. Twenty days of bed rest reduced muscle strength and volume together with tendon stiffness and was associated with slower torque development and prolonged electromechanical delay (46). A subsequent bed-rest study reported reductions in tendon stiffness and increased hysteresis after prolonged unloading (47). Similarly, 14 days of unilateral lower-limb suspension reduced knee-extensor torque, quadriceps cross-sectional area, and patellar-tendon stiffness in healthy men (48). Longer-duration simulated microgravity also decreased the structural and material stiffness of human tendon (49). These findings establish a biologically plausible secondary pathway in which muscle weakness, physical inactivity, or disuse reduces tendon loading and progressively compromises tendon mechanical properties. They do not determine the origin of tendon alterations in every individual with sarcopenia, but demonstrate that reduced loading itself is sufficient to produce coordinated muscle and tendon deconditioning.

Intervention studies further reinforce this integrated view by demonstrating that aged tendon retains substantial mechanosensitivity. In older women, 14 weeks of high-strain cyclic loading increased ankle plantarflexion moment by 22%, augmented tendon stiffness and induced approximately 6% hypertrophy of the free tendon (50). Consistently, broader syntheses indicate that tendon adaptation in older individuals remains robust when mechanical loading is sufficiently high, typically in the range of 80-90% of maximal voluntary contraction or comparable strain magnitudes (11). These findings imply that functional decline of the muscle-tendon unit is not irrevocably fixed but retains a degree of load-dependent plasticity, even in advanced age.

Together, aging and unloading studies support a view of the muscle–tendon unit as an integrated system that exhibits coordinated functional decline while retaining substantial load-dependent plasticity. The observed coordination may arise through intrinsic aging of muscle or tendon, secondary tendon adaptation to altered muscle-generated loading, or parallel responses to shared endocrine–metabolic influences. None of these relationships require direct biochemical communication between the tissues. Accordingly, biomechanical evidence is most appropriately interpreted as establishing functional coupling within this unit, while the extent to which one tissue molecularly instructs the other remains an open question.

Metabolic dysfunction, inflammaging, and vascular insufficiency are not unique to the muscle–tendon unit. Their unit-specific significance arises from the serial organization of muscle, MTJ, and tendon within a single force-transmission pathway and from the different biological responses of these tissues to the same systemic stressors. Muscle and tendon respond differently to these systemic stressors because of their distinct cellular composition, ECM architecture, vascular supply, and mechanical functions. Consequently, aging-associated alterations do not necessarily occur synchronously. Muscle dysfunction may predominate in some individuals, intrinsic tendon or interface remodeling may be more prominent in others, and reduced muscle-generated loading may itself induce secondary tendon deconditioning. Reduced muscle contractility, impaired tendon matrix remodeling, altered tissue stiffness, and compromised myotendinous junction integrity may progressively disrupt the coordination between force generation and force transmission. This mechanical and biological mismatch may help explain why declines in muscle strength and physical performance frequently exceed those predicted by muscle mass loss alone. Thus, the muscle–tendon unit framework emphasizes not the specificity of the upstream systemic stressors themselves, but the functional consequences arising from differential adaptation and impaired coordination between these mechanically coupled tissues.

Taken together, current evidence establishes metabolic dysfunction, inflammaging, vascular insufficiency, and altered mechanical loading as major contributors to skeletal muscle decline, with growing but comparatively less extensive evidence indicating that related processes also compromise tendon homeostasis. For tendon and the MTJ, however, this evidence derives predominantly from aging and experimental studies rather than direct comparisons involving clinically defined sarcopenia. These factors are not specific to the muscle–tendon unit, and their presence alone does not establish unit-level pathology. Their relevance arises from the distinct timing, magnitude, and functional consequences of remodeling in muscle, tendon, and the MTJ. Muscle and tendon may remodel in parallel under shared systemic influences; reduced muscle force and physical activity may drive secondary tendon deconditioning; and intrinsic tendon aging may independently alter the mechanical environment through which muscle force is transmitted. These trajectories can converge on impaired coordination between force generation and force transmission without requiring a uniform sequence of tissue degeneration. Systemic aging therefore provides a permissive pathological context, whereas differential tissue remodeling and failure of mechanical integration constitute the more specific mechanisms through which this context may contribute to sarcopenic functional decline.

## 3 Muscle-to-tendon signaling

### 3.1 Developmental evidence for muscle-dependent tendon specification

The most reliable foundation for muscle-to-tendon signaling derives from embryonic systems (**Figure 2**). In chick limb development, FGF4 expressed at the extremities of muscle is required for maintenance of tendon-associated programs that, in muscle-less or aneural limbs, expression of scleraxis (SCX) and tenascin is markedly reduced, whereas exogenous FGF4 restores their expression (51). Recent studies further reinforce and extend this paradigm. Single-cell and genetic lineage analyses have confirmed that tendon specification and maintenance remain tightly coupled to muscle-derived cues, with TGFβ signaling acting as a central regulator of tendon cell fate and matrix organization in developing musculoskeletal tissues (52, 53). This principle was subsequently refined by work demonstrating that both FGF-ERK/MAPK and TGFβ-SMAD2/3 signaling are necessary and sufficient for SCX expression in chick limb cells, and that muscle contraction is required to sustain tendon differentiation markers, including SCX, TNMD and THBS2. Under immobilization, exogenous FGF4 or TGFβ2 preserves SCX expression, whereas TGFβ2, but not FGF4, also maintains TNMD and THBS2, indicating differential roles of these pathways in maintaining tendon identity (54). These findings place biochemical signaling and mechanical activity within a unified developmental framework that tendon fate is not specified by load alone or by soluble cues in isolation, but by a contraction-linked signaling environment in which muscle functions as an instructive tissue.

**Figure 2:**
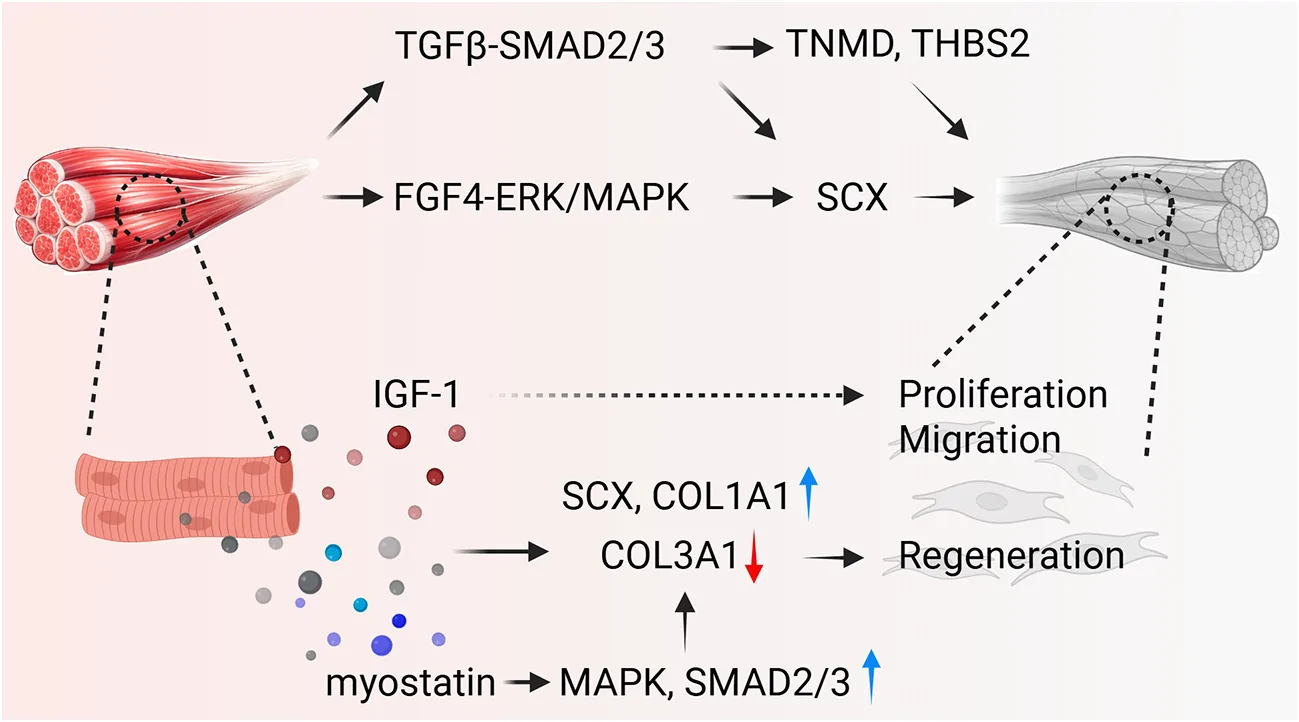
Muscle-to-tendon signaling across developmental and adult contexts. Schematic illustration of muscle-derived signaling pathways regulating tendon specification and tenocyte behavior. Developmental signaling is mediated by FGF-ERK/MAPK and TGFβ-SMAD2/3 pathways that control tendon marker expression. In adult systems, muscle-derived factors, including IGF-1, myostatin, and other secretome components, modulate tenocyte proliferation, migration and matrix-related gene expression in a state-dependent manner.

### 3.2 Muscle state-dependent regulation of tenocytes

Within this section, muscle-to-tendon signaling refers specifically to signal-mediated effects that can be distinguished from obligatory mechanical loading between the two tissues. Accumulating evidence demonstrates that muscle state can reshape tenocyte behavior. The most informative models are those that explicitly uncouple biochemical communication from mechanical continuity, allowing muscle and tendon cells to interact without direct force transmission (55). In such settings, tendon cell responses track the functional state of the muscle compartment, supporting the concept that muscle exports biologically active signals rather than merely imposing mechanical load (**Figure 2**). Transwell-based studies represent a major advance in this regard. Secretome derived from mechanically stimulated myoblasts has been shown to modulate tenocyte phenotype, with moderate loading conditions eliciting the strongest proliferative and migratory responses (56). Mechanistic interrogation further identified IGF-1 as a load-sensitive component of the muscle secretome, and pharmacological inhibition of IGF-1R attenuated the tenocyte response, indicating partial pathway dependence. This is supported by experimental work demonstrating that conditioned media from mechanically stimulated myoblasts enhances tenocyte proliferation and migration via IGF-1 dependent signaling. A subsequent three-dimensional tendon-healing model extended this concept from cell behavior to matrix programming. In engineered tendon constructs, secretome from mechanically stimulated myoblasts enhances SCX and COL1A1 expression while limiting COL3A1 upregulation, consistent with a shift toward a more regenerative phenotype (57). Such effects have been observed in fibrin-based tendon models exposed to muscle-derived conditioned media, which promote tenogenic gene expression and early matrix organization. Although these systems do not establish that identical signaling programs operate quantitatively or causally *in vivo* during sarcopenia, they support a more precise conclusion than was previously possible. Adult muscle-derived soluble factors can modulate tenocyte proliferation, migration and matrix-related gene expression in experimentally decoupled systems, and these effects are conditioned by the mechanical state of the muscle cells that generate the secretome.

### 3.3 Myostatin as an entangled signaling candidate

Myostatin emerges as a catabolic indicator of muscle-to-tendon signaling (58). A factor that restrains muscle growth and is elevated in catabolic states could plausibly coordinate tendon adaptation to altered muscular demand. Experimental evidence supports a functional role for myostatin in tendon biology (**Figure 2**). In myostatin-deficient mice, tendons exhibit reduced size, cellularity and mechanical integrity, accompanied by decreased expression of collagen I, SCX and tenomodulin. Conversely, myostatin stimulation activates MAPK and SMAD2/3 signaling in tendon fibroblasts, promoting proliferation and tenogenic gene expression (59). The tenogenic potential of myostatin extends beyond canonical tendon fibroblasts. In C2C12 myoblasts, myostatin suppresses myotube formation while inducing a spindle-shaped, tenomodulin-positive phenotype through SMAD3-dependent signaling, while pharmacological inhibition of ALK receptors or suppression of SMAD3 attenuates this transition (60). This indicates myostatin can influence not only tendon-resident cells but also lineage-plastic myogenic populations, linking muscle-state signaling to broader cell-fate regulation. However, despite this biological plausibility, myostatin remains interpretively complex as a defined mediator in adult sarcopenia. Myostatin-deficient models profoundly alter muscle size, loading history and developmental patterning, making it difficult to disentangle direct paracrine or endocrine effects on tendon from secondary biomechanical consequences (59). Accordingly, myostatin is best viewed not as a definitively established directional regulator *in vivo*, but as a biologically persuasive candidate whose causal position within an adult muscle-to-tendon signaling axis remains experimentally entangled.

### 3.4 Extracellular vesicles as potential candidates

A further candidate layer of muscle-to-tendon communication is extracellular-vesicle transfer. Conceptually, EVs provide a plausible mechanism by which muscle could export state-dependent molecular information to adjacent connective tissue. This possibility is particularly relevant in aging, where muscle secretory programs and microRNA profiles undergo substantial remodeling. A well-characterized example is miR-29, which is upregulated in aged skeletal muscle and suppresses IGF-1 to impair progenitor proliferation and promote senescent responses (27). Although not demonstrated in a tendon-specific context, this finding illustrates how aging muscle can package biologically potent regulatory signals into a paracrine framework with potential relevance to tissue remodeling. Direct evidence for EV-mediated muscle-to-tendon signaling remains limited. Proteomic analysis of extracellular vesicles derived from primary human tendon and muscle fibroblasts demonstrates marked differences in cargo composition, with tendon-derived EVs enriched for ECM-associated proteins, including TGFβ1, whereas muscle-derived EVs are relatively enriched for proteins linked to muscle-associated and matrix-remodeling functions (16). These observations support the biological plausibility of vesicle-based inter-tissue communication within the muscle-tendon unit, but they do not establish that muscle-derived EVs carrying defined cargo reach tendon *in vivo* during aging and impose a specific phenotypic outcome. EVs and their RNA cargo are therefore best regarded as a high priority but still immature mechanistic hypothesis.

Taken together, current evidence demonstrates that muscle can influence tendon biology through more than mechanical loading alone. Developmental studies establish muscle-dependent regulation of tendon specification, whereas conditioned-medium and mechanically decoupled systems show that adult muscle-derived factors can modify tenocyte proliferation, migration, and matrix-related gene expression. Candidate mediators include IGF-1, myostatin-related signaling, and extracellular vesicle cargo, although their relative contributions remain incompletely defined. The strength of evidence nevertheless differs across biological levels. Direct signaling capacity is well supported in developmental and reductionist experimental systems, while tissue-specific *in vivo* evidence demonstrating that muscle-derived factors regulate tendon aging independently of loading, vascular supply, and systemic endocrine influences remains lacking. Muscle-to-tendon signaling is therefore experimentally supported, but direct evidence from clinically defined sarcopenia remains insufficient to determine its quantitative contribution to tendon dysfunction.

## 4 Emerging evidence for tendon-to-muscle regulation

### 4.1 Interface and matrix-mediated bases for tendon influence

In contrast to muscle-to-tendon signaling, evidence supporting direct biochemical tendon-to-muscle communication in adult systems remains limited and is largely derived from reductionist models. At present, available evidence primarily demonstrates the capacity of tendon-derived cues to influence myogenic behavior under experimental conditions, rather than establishing a physiologically validated endocrine or paracrine regulatory network *in vivo*. Tendon-to-muscle signaling is biologically plausible because muscle and tendon are linked not only mechanically but also through a highly specialized interface. The myotendinous junction (MTJ) provides a local environment in which ECM organization, adhesion systems, and mechanically responsive signals may shape cellular behavior on both sides of the interface (24, 61). Tendon-derived influence on muscle need not be restricted to classical soluble mediators, but may also arise through matrix-associated or interface-dependent mechanisms. In the context of sarcopenia, this possibility is particularly relevant, as aging-related alterations in tendon structure and niche function may modify the local signals experienced by adjacent muscle (62). The following evidence therefore addresses experimentally demonstrable tendon-derived influences on myogenic cells and engineered interfaces, rather than an established tendon-to-muscle signaling axis *in vivo*.

### 4.2 Tendon-derived cues bias myogenic differentiation

Direct evidence supporting tendon-to-muscle signaling in adult systems remains limited but is emerging from human and mammalian co-culture models. Tendon-derived cells modulate myogenic differentiation in a selective and non-mitogenic manner. In primary human co-culture systems, tendon fibroblasts enhance myotube formation without increasing myoblast proliferation, as evidenced by unchanged Ki67 and BrdU incorporation alongside increased fusion index and myotube size (63) (**Figure 3**). This selective effect supports a role for tendon-derived cues in myogenic differentiation and maturation under co-culture conditions, rather than a nonspecific increase in cell growth. The study did not identify a dominant soluble mediator, determine whether direct cell contact was required, or establish that the same effect occurs in adult muscle *in vivo*. More recent engineered human MTJ models further support the biological plausibility of such inter-tissue regulation by showing that co-culture of human myoblasts and tenocytes, especially under cyclic strain, enhances MTJ marker gene expression and promotes anisotropic myocyte-tenocyte integration capable of force transmission (64). These models provide a controlled platform for studying interfacial cell–cell and cell–matrix interactions, but do not independently demonstrate a physiologically active tendon-derived signaling pathway in sarcopenia.

**Figure 3:**
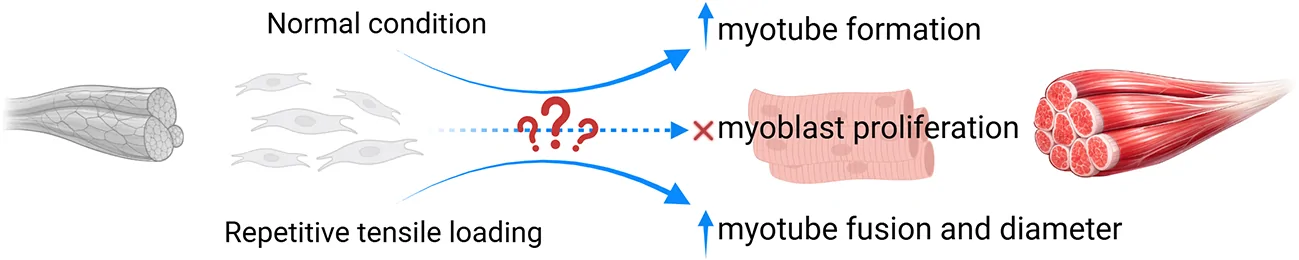
Tendon-derived regulation of myogenic differentiation. Schematic illustration of tendon-derived cues influencing myogenic progression. Tendon-derived signals selectively promote myotube formation without altering myoblast proliferation, while repetitive tensile loading further enhances myotube fusion and diameter. Despite consistent phenotypic effects across experimental systems, the underlying mediators and signaling pathways remain incompletely defined.

### 4.3 Mechanical state shapes tendon-derived signaling output

A critical extension of the current evidence is that tendon-derived influence on myogenic cells is mechanosensitive. In tissue-engineered human tendon constructs, repetitive tensile loading altered the biological activity of tendon-conditioned medium, which was able to increase myotube fusion and diameter without affecting myoblast proliferation, while reducing myostatin, MYH7 and AXIN2 expression in recipient myotubes (65) (**Figure 3**). Since the tendon construct and recipient myogenic cells were physically separated, this study provides the clearest current evidence that mechanically conditioned tendon-derived soluble factors can modify myotube formation *in vitro*. Support for this interpretation comes from engineered interface systems showing that co-culture of myoblasts and tenocytes under strain enhances MTJ-associated gene expression and promotes structured myocyte-tenocyte integration, indicating that inter-tissue signaling is amplified by the combined effects of cellular juxtaposition and mechanical stimulation (64). However, the active mediator or mediator class remains unidentified, and neither model establishes the tissue origin, distribution, physiological concentration, or functional necessity of such signals *in vivo*. Current findings therefore demonstrate a mechanically regulated tendon-to-muscle signaling capacity in engineered systems, while its relevance to muscle homeostasis and sarcopenia in intact organisms remains unresolved.

### 4.4 Aging-related changes in the tendon signaling environment

Aging tendon is increasingly characterized by stem/progenitor cell dysfunction, impaired regenerative capacity, altered niche signaling, and defective cell-matrix interactions, alongside reduced migratory potential and diminished healing competence (66, 67). The tendon niche is now understood to encompass not only resident cells, but also ECM composition, soluble mediators, oxidative stress, vascular inputs, and systemic metabolic signals, forming a multi-layered regulatory environment (68). These age-related changes establish that the biological state of tendon is not preserved during aging, but they do not by themselves demonstrate altered signaling to skeletal muscle.

Structural studies indicate that aging is accompanied by changes in interfascicular matrix organization and reduced capacity for fascicle sliding, which may alter local force transmission and matrix-mediated signaling environments (69). In parallel, broader syntheses of tendon biology emphasize that aging affects not only mechanical properties but also cell–matrix interactions and the regulatory microenvironment governing tissue homeostasis and repair (70). These changes collectively raise the possibility that aging tendon provides a qualitatively altered local signaling milieu to adjacent muscle. Consistent with this possibility, tendon-derived extracellular vesicles exhibit a protein-cargo profile distinct from that of muscle-fibroblast-derived vesicles, including relative enrichment of ECM-associated proteins and TGFβ1 (16). However, it remains unknown whether these vesicles or other tendon-derived factors reach adjacent myofibers *in vivo* or exert biologically meaningful effects on muscle homeostasis during aging.

The current evidence therefore supports a testable hypothesis: aging-related changes in tendon cells, ECM, and mechanically conditioned secretory output may alter the local environment encountered by adjacent muscle. Demonstrating this relationship will require identification of tendon-derived mediators, confirmation of their transfer to muscle *in vivo*, and tendon-specific perturbation showing a muscle phenotype independent of changes in mechanical loading. Until these criteria are met, aging tendon should be considered a plausible source of altered local cues, not an established regulator of sarcopenic muscle remodeling.

Taken together, the available evidence supports an experimental capacity for tendon-derived cells, conditioned media, and interface-associated matrices to influence myogenic differentiation and organization. The strongest evidence comes from human co-culture and mechanically loaded tendon-construct models, in which tendon-derived inputs enhance myotube formation without stimulating myoblast proliferation. Evidence decreases substantially at higher levels of biological organization. The responsible mediators remain unidentified, tendon-derived extracellular vesicle transfer to muscle has not been demonstrated *in vivo*, and no tendon-specific perturbation has established a causal effect on muscle homeostasis or metabolism during aging or sarcopenia. Tendon-to-muscle regulation should be regarded as an emerging and experimentally testable mechanism, not yet as a validated component of a robust bidirectional signaling network in adult tissue, and its contribution to clinically defined sarcopenia remains unknown.

## 5 The myotendinous junction as a regulatory interface in sarcopenia

### 5.1 Structural and molecular specialization of the MTJ

The MTJ exhibits a level of structural and molecular specialization incompatible with any reductionist view of it as a static anchoring site. It is organized as a deeply folded, interdigitated interface in which sarcolemma projections extend into the tendon matrix, markedly expanding the effective contact area between the two tissues (71). This architecture is not incidental but represents a specialized structure for force transmission across a mechanically heterogeneous interface (72). By amplifying contact area on the order of magnitude of 10–20 folds, the MTJ distributes mechanical stress and reduces focal strain concentration during repeated loading, thereby enabling efficient force transmission under conditions of high mechanical demand. Notably, the MTJ is molecularly distinct from both muscle and tendon. Its ECM is not a passive mixture of the two compartments, but a junction-enriched microenvironment characterized by specific matrix components, including collagen XXII and periostin, which are selectively enriched at the MTJ (73). These features are consistent with a region optimized not only for mechanical resilience but also for localized mechanobiological regulation.

Recent proteomic studies further reinforce the view that the MTJ constitutes a compositionally defined molecular domain. In human semitendinosus MTJ samples, deep proteomic profiling quantified more than 3, 000 proteins and identified over 100 significantly enriched MTJ proteins, including previously unrecognized markers such as CD151, FERMT2, CILP and ITGA10 (23). Importantly, spatial proteomic analyses further show that many proteins exhibit peak expression specifically at the MTJ, highlighting the interface as a region of distinct molecular identity and high network connectivity (74). These findings make it difficult to interpret the MTJ as a purely structural seam. Instead, the MTJ emerges as a spatially and molecularly defined interface domain, whose architecture and composition are jointly optimized for mechanical transmission and localized biological regulation. This dual specialization provides a necessary foundation for considering it not only as a site of force transfer, but as a candidate regulatory niche within the muscle-tendon unit.

### 5.2 The MTJ as the spatial framework for inter-tissue signaling

At the MTJ, mechanical integration and biochemical communication should be considered related but distinct processes. One of the recurring weaknesses of inter-tissue signaling studies is that communication is often discussed in molecular terms without being anchored in tissue architecture. The MTJ addresses this problem since it is the site of greatest local integration between muscle and tendon, where force transmission, matrix organization and cell behavior converge. Under this circumstance, it provides the most plausible spatial framework in which biochemical signals, matrix-derived cues and mechanically induced phenotypic changes could be coordinated and converted into durable structural outcomes (75). Developmental and mechanobiological studies strongly support this interpretation. In zebrafish, the onset of muscle contraction promotes the formation of elongated, microtubule-rich tenocyte projections along the MTJ, whereas paralysis shortens these projections, reduces adjacent ECM and decreases SMAD3 phosphorylation (76). These findings localize force-dependent TGFβ signaling to the interface itself and show that MTJ-associated tendon-cell morphology and matrix deposition are modulated by contractile input rather than passively maintained by tissue proximity. Mouse developmental work extends this principle by showing that MTJ assembly is load dependent at the level of matrix patterning and maturation (77). MTJ-specific ECM deposition is initiated under static loading associated with growth, but cyclic loading is required for development of mature interdigitated morphology. In the absence of normal muscle contractility, MTJ-enriched proteins including COL22A1 and THBS4 are markedly diminished, together with reductions in tendon-associated matrix proteins such as TNXB and FMOD. These data highlight that MTJ identity is not simply a by-product of anatomical adjacency between muscle and tendon, but the output of a mechanically regulated molecular program.

This framework also highlights that inter-tissue regulation at the MTJ may extend beyond classical soluble-factor signaling. The relevant information may need to be encoded in matrix composition, ligand presentation, stiffness, basement-membrane organization and cell-matrix adhesion. Consistent with this view, engineered interface studies show that tissue-specific ECM hydrogels, particularly tendon-derived ECM, increase expression of the canonical MTJ-associated protein paxillin, and that collagen XXII is likewise favored in interface-promoting matrix environments (78, 79). These findings identify the MTJ as a plausible spatial context in which mechanical, matrix-associated, and soluble cues may be integrated, while direct adult *in vivo* evidence for bidirectional biochemical signaling across this interface remains unavailable.

### 5.3 Age-dependent remodeling of the MTJ

Although a characteristic MTJ phenotype has not yet been established in clinically defined sarcopenia, aging studies demonstrate that this interface undergoes substantial structural and cellular remodeling. In mouse soleus, aging induces pronounced remodeling of the junction, including an apparent ~3.5-fold increase in MTJ profiles per cross-section. This remodeling is accompanied by a ~31% increase in proximal tendon length and increased collagen deposition between 6 and 24 months of age, indicating that the aging MTJ undergoes active structural reorganization rather than passive loss (80). Importantly, more recent work suggests that this structural remodeling is coupled to cellular-level alterations within the MTJ niche itself. High-resolution 3D analyses demonstrate that aging leads to expansion of the MTJ interface area together with reduced vascularity and increased cellular senescence, including marked accumulation of p16^+^ cells within COL22A1-positive MTJ-specific populations (81). These findings extend the concept of MTJ remodeling beyond geometry, indicating that structural changes are accompanied by declining biological fitness of interface-resident cells. The biological meaning of this remodeling remains unresolved and should not be oversimplified. On the one hand, expansion of the fiber-termination area may represent a compensatory adaptation, potentially preserving force transmission under conditions of declining muscle quality or altered tendon mechanics. On the other hand, the same changes may reflect matrix dysregulation, as aging across musculoskeletal tissues is frequently associated with altered collagen homeostasis, including accumulation, fragmentation, and impaired turnover, which can lead to increased stiffness without improved mechanical efficiency (82).

A critical implication is that MTJ remodeling occurs against a background of intrinsic matrix remodeling processes, in which both excessive collagen deposition and impaired matrix turnover can coexist. This creates a scenario in which increased matrix content does not necessarily translate into improved functional performance, but may instead reflect a shift toward a stiffer, less adaptive interface environment. At present, the evidence is insufficient to classify the aged MTJ as either adaptive or degenerative. A more defensible interpretation is that aging induces interface remodeling whose functional consequences are context-dependent, shaped by matrix composition, cellular state and mechanical loading conditions. This nuance is particularly important for sarcopenia. Age-related remodeling of the MTJ may modify the functional expression of muscle weakness by altering the interface through which contractile force is transmitted. Changes in interface properties could therefore contribute to the sarcopenic phenotype by linking mechanics, matrix biology, and inter-tissue signaling within a single spatial domain. Despite this strong conceptual rationale, direct functional studies remain scarce, still lacking robust *in vivo* evidence defining how MTJ remodeling alters force-transfer efficiency, local signaling environments, muscle regeneration or tendon adaptation, leaving the physiological significance of this remodeling largely unresolved.

### 5.4 The MTJ as a mechanobiological amplifier

The MTJ is particularly relevant to sarcopenia since it represents the site at which multiple age-related processes converge. The specificity of this interface does not derive from the upstream stressors themselves, but from their convergence on a spatially restricted structure responsible for transferring force between tissues with different mechanical and biological properties. Mitochondrial decline, inflammaging, ECM stiffening, tendon matrix degradation and altered loading patterns may arise upstream in muscle, tendon or the systemic environment, but their consequences are likely to be integrated and amplified at the interface. In this case, the MTJ may function as a mechanobiological amplifier, whereby relatively modest impairments in muscle or tendon become disproportionately consequential when transmitted through a structurally specialized interface (72).

Notably, MTJ integrity is mechanically regulated. Experimental work shows that loss of muscle contraction leads to reduced MTJ-specific ECM deposition and impaired maturation of the interface, whereas cyclic loading is required for development of a fully functional MTJ architecture (77). This indicates that the MTJ operates as a load-dependent regulatory site, in which mechanical input directly shapes matrix composition and structural organization. Within this context, age-related alterations in loading patterns and matrix turnover are likely to be integrated at the interface rather than remaining confined to individual tissues. These observations support a model in which the MTJ acts as a convergence point for mechanical and biochemical dysfunction. Alterations in matrix composition, impaired mechanotransduction and disrupted cell-matrix interactions may combine to reduce the efficiency of force transfer and compromise local signaling environments. In this scenario, the aged MTJ may remain structurally present but become functionally compromised, amplifying dysfunction across the muscle-tendon unit. This perspective provides a mechanistic explanation for a long-standing observation in sarcopenia, as muscle strength declines often exceed the loss of muscle mass alone. This discrepancy may arise in part from failure of force transmission and interface-level integration, linking matrix biology, mechanics and inter-tissue signaling within a single spatial domain.

Taken together, positioning the MTJ as a regulatory niche broadens the analysis of sarcopenic weakness from muscle quantity alone to the coordination of force generation and transmission across the muscle–tendon unit. Declines in strength, performance, and adaptability may reflect varying contributions from muscle dysfunction, tendon remodeling, and loss of interface-level integration. This perspective may help explain part of the functional heterogeneity observed among older individuals with comparable reductions in muscle mass or strength. Whether a reproducible pattern of MTJ remodeling specifically distinguishes sarcopenia from physiological aging, however, remains to be determined. It also suggests that the therapeutic relevance of the MTJ is likely to be context dependent: interface-directed strategies may be most valuable when impaired force transmission constitutes a measurable component of the functional deficit. Muscle-focused treatment remains central, while assessment of tendon and MTJ function may help determine whether additional tissue-specific intervention is warranted. Accordingly, incorporating MTJ biology into sarcopenia research may help clarify how interface-level changes contribute to functional decline in selected biological and clinical contexts.

## 6 Therapeutic implications

### 6.1 Exercise as a unit-targeted intervention

At present, exercise remains the most consistently supported intervention for sarcopenia, with resistance-based programs demonstrating reproducible benefits across muscle strength and functional performance. Recent systematic reviews and meta-analyses confirm that resistance training significantly improves handgrip strength, gait speed and overall physical performance in older adults with sarcopenia (83, 84). These findings have established resistance exercise as the clinical cornerstone of sarcopenia management.

However, from the perspective of the muscle-tendon unit, the therapeutic relevance of exercise extends beyond muscle hypertrophy or neuromuscular activation. Mechanical loading simultaneously engages multiple tissues, including muscle, tendon and MTJ, activating a distributed mechanobiological system (85). In skeletal muscle, resistance exercise induces well-characterized anabolic and metabolic adaptations, including enhanced protein synthesis, improved mitochondrial function and increased neuromuscular efficiency (86–88). Meanwhile, tendon demonstrates load-dependent remodeling characterized by improvements in stiffness, material properties and collagen organization, even in older individuals (89, 90). Notably, these adaptations are coordinated. Human biomechanical and intervention studies consistently show that improvements in muscle strength occur in parallel with changes in tendon mechanical properties, suggesting that functional recovery reflects recalibration of the entire muscle-tendon unit (10). This coordination is mechanistically significant since force expression *in vivo* depends not only on muscle contractile capacity, but also on the efficiency with which that force is transmitted through tendon. Consistent with this view, human studies in older adults demonstrate that age-related reductions in rapid torque generation are attributable not only to slower muscle contractile behavior, but also to tendon-related mechanical factors that delay or attenuate force transmission (12). Reduced Achilles tendon stiffness has also been shown to disrupt calf muscle neuromechanics and increase the metabolic cost of walking, indicating that tendon dysfunction can degrade movement efficiency even when muscle tissue remains present and functional (91, 92).

An ECM-centered view further implies how exercise exerts its effects at the level of the muscle–tendon unit. Resistance exercise has been shown to directly modulate ECM composition and organization in muscle, including increased collagen turnover, remodeling of perimysial and endomysial networks, and improved matrix alignment with contractile fibers (93), which in turn enhances lateral force transmission efficiency and the mechanical expression of contractile force (94). Parallel observations are evident in tendon. Exercise interventions in older adults consistently show improvements in tendon stiffness and biomechanical behavior (11, 50), which are closely linked to load-induced collagen reorganization and matrix remodeling, suggesting that exercise targets tendon function primarily through ECM-mediated mechanisms. These adaptations are not confined to individual tissues but influence the behavior of the integrated unit, which may provide a plausible explanation for why exercise can improve strength and performance without large increases in muscle mass. Conversely, persistent ECM dysfunction, either in intramuscular connective tissue, tendon matrix, or the MTJ microenvironment, may limit the functional translation of otherwise adequate muscle adaptation. From this perspective, exercise acts, at least in part, as an ECM-targeting intervention. By restoring matrix quality, alignment and mechanotransductive capacity across the muscle-tendon unit, mechanical loading improves force generation, transmission and integration. In the context of sarcopenia, this suggests that therapeutic strategies should not focus solely on increasing muscle mass, but also on restoring ECM integrity and remodeling capacity across the muscle-tendon unit, as these factors are likely to determine whether structural gains can be effectively translated into functional improvement.

This integrated view also helps explain why pharmacological or nutritional gains in muscle mass do not consistently translate into proportional improvements in strength or mobility (95, 96). When the mechanical behavior of tendon is not appropriately matched to the force-generating capacity of muscle, or when ECM organization or MTJ function is compromised, increases in muscle capacity may be only partially expressed at the level of movement. These observations raise the possibility that interventions capable of coordinating muscle adaptation with tendon mechanics and matrix-mediated force transmission may produce more complete functional gains, although this proposition has not yet been tested directly in sarcopenia. This interpretation is aligned with recent position statements emphasizing that exercise prescriptions for sarcopenia should be structured, progressive and biologically specific, rather than generalized increases in physical activity (97, 98).

The practical implication of this framework is not simply to prescribe more exercise, but to consider whether future exercise programs should evaluate loading according to the adaptive requirements of both muscle and tendon. A conventional muscle-centered program may be sufficient when reduced contractile capacity is the predominant limitation. However, when tendon compliance, delayed force transmission, or a discrepancy between gains in muscle strength and physical performance is identified or suspected, future studies should determine whether modification of loading magnitude, contraction duration, progression rate, or recovery period can promote tendon adaptation alongside muscle strengthening (50, 99). The muscle–tendon unit framework therefore refines the therapeutic question by asking which loading characteristics are required, which component is limiting functional recovery, and whether adaptation in one tissue is being translated into improved movement.

The muscle–tendon unit framework is not intended to replace exercise-based interventions, which remain the foundation of sarcopenia management. Instead, it provides a broader mechanistic context for optimizing rehabilitation strategies. Muscle and tendon exhibit distinct biological responses and temporal patterns of adaptation to mechanical loading, suggesting that interventions designed primarily to maximize muscle hypertrophy may not necessarily optimize tendon remodeling or MTJ integrity. Therefore, considering the coordinated adaptation of the entire muscle–tendon unit may facilitate loading strategies tailored to tissue-specific adaptation rates, monitoring of whether strength gains translate into improved force transmission and physical performance, and future development of tissue-specific therapeutic approaches. Thus, the innovation of the muscle–tendon unit framework lies less in replacing existing therapies than in broadening the biological and functional outcomes considered when evaluating those therapies.

### 6.2 Load magnitude and progression influence tendon adaptation

Tendon adaptation is highly dependent on the magnitude and duration of mechanical loading, and not all exercise is biologically equivalent in this context. Studies conducted primarily in healthy or otherwise non-sarcopenic populations suggest that tendon adaptation is favored by sufficiently high mechanical strain, with commonly cited experimental ranges of approximately 4.5–6.5% strain and loading intensities approaching 80–90% of maximal voluntary contraction (100). These values should not be interpreted as validated prescription thresholds for individuals with sarcopenia. Lower mechanical doses have generally produced smaller or less consistent changes in tendon mechanical properties in experimental and training studies (101). High-strain cyclic loading can increase tendon stiffness and material properties in older adults, supporting the preservation of tendon mechanosensitivity with aging (50). Conversely, lower-intensity or insufficiently dosed loading may produce limited or inconsistent tendon adaptation (99). The clinically appropriate loading magnitude for sarcopenic populations therefore remains uncertain and is likely to depend on muscle strength, tendon mechanical behavior, functional status, comorbidity burden, and individual loading tolerance. However, a tendon-oriented rehabilitation strategy should not be equated with eccentric exercise alone. Clinical studies in tendinopathy indicate that both eccentric and heavy slow resistance loading can produce sustained improvements, suggesting that tendon-directed adaptation can be achieved through different contraction modes when loading magnitude and progression are appropriately controlled (102). These findings cannot be directly extrapolated to sarcopenia, but they illustrate that tendon-specific loading is defined by the mechanical stimulus rather than by a single exercise modality.

Tendon adaptation follows a distinct temporal and mechanobiological profile compared to muscle. Whereas skeletal muscle responds relatively rapidly to loading through hypertrophy and neural adaptation, tendon exhibits slower, more threshold-dependent remodeling kinetics, with changes emerging over weeks to months (11, 103). This divergence reflects differences in tissue turnover, cellular activity and matrix remodeling dynamics, and implies that muscle and tendon do not adapt synchronously under identical training conditions. The nature of this adaptation is also qualitatively distinct. Improvements in tendon function are driven predominantly by changes in material properties such as stiffness and modulus, rather than by large increases in cross-sectional area (11, 99). As a result, tendon remodeling may be functionally substantial but morphologically subtle, making it easy to underestimate when assessed using size-based metrics alone.

These observations provide a mechanistic rationale for considering tendon adaptation in future sarcopenia exercise studies, but they do not yet establish a sarcopenia-specific loading prescription. Low-intensity or insufficiently progressed exercise may improve general mobility, although whether such loading is insufficient for tendon adaptation in individuals with sarcopenia has not been directly established. Conversely, imposing high loads without accommodating the slower and less linear adaptation kinetics of tendon may increase the risk of overload and poor adherence in older individuals. Rather than defining a fixed muscle–tendon-oriented exercise protocol, current evidence supports testing whether loading progression can be individualized according to muscle capacity, tendon mechanical responses, functional gains, and tolerance. Such studies will be needed to determine whether coordinated adaptation of muscle and tendon provides functional benefits beyond conventional resistance-training approaches.

For individuals unable to tolerate conventional high external loads, low-load resistance training combined with blood-flow restriction represents a potential alternative for investigation. Studies in younger or healthy adults have reported Achilles and patellar tendon adaptations comparable to those achieved with high-load resistance training, but its safety, feasibility, and effectiveness in older adults with sarcopenia remain to be established (104, 105).

The muscle–tendon unit perspective may also refine nutritional support by distinguishing interventions aimed primarily at muscle protein accretion from adjuncts intended to support connective-tissue remodeling. Adequate dietary protein and essential amino acids remain central to muscle adaptation, whereas collagen- or gelatin-derived peptides, particularly when combined with vitamin C and mechanical loading, have been investigated for their potential to enhance collagen synthesis and tendon adaptation (106–108). However, current evidence has not established that these strategies improve tendon mechanics, MTJ function, or clinical outcomes in older adults with sarcopenia. Therefore, they may be considered experimental adjuncts to appropriately prescribed exercise rather than substitutes for established nutritional care or resistance training.

### 6.3 Pharmacological strategies remain incomplete at the unit level

Currently, no drug is approved specifically for sarcopenia, and contemporary reviews and clinical practice guidance continue to place resistance exercise and nutritional support at the center of management (109, 110). This limitation is not simply a failure of drug development; it reflects a mismatch between what many candidate agents improve and what integrated physical function actually requires. Pharmacological approaches targeting anabolic pathways, including testosterone-related therapy, SARMs, and myostatin/activin-pathway inhibition, can increase lean mass and improve strength outcomes, but translation into robust functional benefit has been inconsistent. A meta-analysis of testosterone supplementation in middle-aged and older men reported improvements in muscle mass and some sarcopenic parameters, but not a uniform restoration of physical performance (111). Likewise, enobosarm increased lean body mass and improved physical function in some settings, but these effects were not sufficient to establish SARMs as a clinically definitive solution for sarcopenia (112, 113). The myostatin/activin axis is particularly instructive. In older weak fallers, LY2495655 increased appendicular lean mass but did not produce a uniformly convincing functional rescue across the population studied (114). In a phase II proof-of-concept study, bimagrumab increased muscle mass and strength and improved mobility in slower walkers (115), yet in a subsequent randomized clinical trial in community-dwelling older adults with sarcopenia, bimagrumab did not significantly outperform optimized standard care on the primary functional endpoint (116). This pattern is biologically informative: increasing muscle quantity is feasible, whereas restoring whole-body performance is considerably more difficult. These discrepancies indicate that changes in muscle quantity alone do not fully account for treatment-related changes in mobility. Whether persistent limitations in tendon mechanics, force transmission, neuromuscular function, or other systemic factors contribute to this incomplete translation has not been established and should be evaluated directly in future trials. The implication is not that pharmacology is unimportant, but that future drug development in sarcopenia should incorporate functional outcomes that capture the translation of muscle gains into mobility, in addition to conventional measures of muscle mass and strength. The muscle–tendon unit perspective further highlights the need to distinguish agents that increase contractile tissue quantity from interventions that improve the functional expression of that capacity. At present, no tendon- or MTJ-specific pharmacological intervention has been validated for sarcopenia. Future pharmacological studies should therefore evaluate muscle strength and quantity together with tendon mechanical properties, force-transmission behavior, and physical performance before claiming unit-level therapeutic efficacy.

### 6.4 Metabolic and vascular optimization as enabling strategies

The shared-driver framework also has therapeutic consequences. In skeletal muscle, mitochondrial dysfunction is commonly considered as a central component of sarcopenic decline, with implications for ATP supply, proteostasis, redox control, and the capacity to adapt to repeated loading (117, 118). This is one reason exercise remains uniquely effective, as it improves mitochondrial energetics and quality control in addition to providing mechanical loading. Likewise, microvascular rarefaction and impaired capillary support constrain metabolism, fitness, and functional capacity in older adults (119). Sarcopenic elders show lower skeletal muscle capillarization, and this feature has been linked to reduced exercise capacity (120). Further studies show age-associated alterations in capillary proximity to satellite cells and reductions in capillary supply to muscle fibers, reinforcing the idea that impaired vascular support can limit regenerative and adaptive potential (121, 122).

This constraint is not confined to muscle but extends to the muscle-tendon unit through limitations in the local regulatory niche that governs tissue adaptation. Although tendon is intrinsically hypovascular, its capacity for maintenance and remodeling remains dependent on a permissive microenvironment. Aging is associated with a decline in tendon stem/progenitor cell function (44), accompanied by reduced matrix production and impaired three-dimensional tissue organization arising from both cell-intrinsic defects and ECM dysregulation (123). These alterations coincide with increased oxidative stress, which further disrupts matrix homeostasis and compromises tendon remodeling capacity (124). Under these conditions, the biological competence of tendon to respond to mechanical loading is diminished, even when an adequate external stimulus is applied. Metabolic and vascular conditions therefore set the boundary within which loading-induced adaptation can occur, determining whether improvements in muscle function can be effectively transmitted through the muscle-tendon unit. Accordingly, correction of metabolic, inflammatory, nutritional, or vascular constraints should be viewed as an enabling strategy that may improve responsiveness to exercise, rather than as an independent substitute for appropriately prescribed mechanical loading.

### 6.5 Targeting the MTJ as an emerging therapeutic concept

A direct implication of the muscle-tendon unit framework is that the MTJ may represent a functionally relevant site of impairment in selected individuals with sarcopenia. Although current interventions are not specifically designed to restore force-transfer efficiency at this interface, strategies that influence ECM organization, cell-matrix adhesion, and local mechanotransductive signaling have the potential to improve the efficiency with which contractile force is transmitted across the muscle-tendon unit. At present, mechanically guided rehabilitation represents the most clinically plausible means of influencing MTJ adaptation. Other matrix-directed or mechanotransductive strategies remain experimental, and no MTJ-specific intervention has been validated for sarcopenia. The MTJ should therefore be regarded as a candidate site for future therapeutic development rather than an established treatment target.

Taken together, these considerations shift the therapeutic focus from isolated tissue-level improvements to the coordination of function across the muscle-tendon unit. The relative therapeutic importance of muscle capacity, metabolic and vascular support, tendon mechanics, and interface integrity is likely to vary according to the dominant source of functional limitation. In many individuals, muscle-directed exercise, nutritional support, or pharmacological intervention may remain the principal therapeutic priority. In others, a mismatch between muscle capacity and tendon mechanical behavior or impaired force transmission may limit the functional expression of improved muscle capacity and justify further evaluation of whether loading magnitude, progression, recovery, or treatment monitoring should be modified, while more specific interface-directed interventions remain investigational. Identifying the predominant site of dysfunction may therefore be more informative than assuming that every component of the muscle–tendon unit requires equivalent intervention.

### 6.6 Clinical assessment and potential measurement approaches

Current clinical diagnosis of sarcopenia should continue to follow established consensus criteria. EWGSOP2 prioritizes low muscle strength as the principal indicator of probable sarcopenia, uses low muscle quantity or quality to confirm the diagnosis, and defines poor physical performance as an indicator of severe sarcopenia (125). AWGS 2019 similarly evaluates muscle mass, muscle strength, and physical performance, while also providing a “possible sarcopenia” pathway suitable for community and primary-care settings (126). Accordingly, tendon or MTJ measurements should not currently be regarded as diagnostic requirements for sarcopenia. Their potential value lies instead in complementary functional phenotyping, particularly in individuals whose mobility impairment appears disproportionate to the measured loss of muscle mass or strength.

Conventional B-mode ultrasonography provides the most accessible starting point for assessing components of the muscle–tendon unit. It can characterize tendon thickness, cross-sectional area, echogenicity, and gross morphology, while dynamic ultrasound can track MTJ displacement during contraction and estimate tendon elongation. Recent automated approaches have improved the speed and reproducibility of MTJ identification and displacement tracking by reducing reliance on manual frame-by-frame analysis (127, 128). These methods are non-invasive and compatible with repeated measurements, but MTJ displacement or tendon morphology alone should not be interpreted as direct measures of interface integrity or force-transfer efficiency.

Shear-wave elastography offers a complementary approach by estimating shear-wave velocity or an apparent elastic property within tendon tissue (129). Studies in older adults indicate that Achilles tendon shear-wave measurements are technically feasible and may detect age-related differences in tendon mechanical behavior (130). However, shear-wave-derived values are not equivalent to mechanical stiffness calculated from tendon force–elongation relationships. Measurements are influenced by ankle position, loading state, acquisition protocol, probe configuration, and analysis method, limiting direct comparison across devices and studies (131). Consequently, shear-wave elastography should currently be interpreted as a research-oriented measure of tendon material behavior rather than a validated diagnostic test for sarcopenia.

More direct estimation of tendon mechanical stiffness or force-transmission behavior generally requires integration of ultrasound with dynamometry or joint-torque measurements. Importantly, tendon stiffness should not be interpreted as an isolated index of tendon quality; its functional significance depends on its relationship to muscle force-generating capacity, tendon elongation behavior, and the efficiency with which force is transmitted across the muscle–tendon unit. In these approaches, tendon force is estimated from joint torque and moment-arm assumptions, while ultrasound is used to quantify tendon elongation or MTJ displacement during contraction. Electromyography and biomechanical modeling may also be needed to account for antagonist coactivation, muscle recruitment, and tendon geometry. Such protocols provide richer information regarding force generation and transmission, but they are more time-consuming, technically demanding, and operator dependent than routine grip-strength or gait-speed testing. They are therefore better suited to mechanistic studies and specialist assessment than to population-level screening at present.

Before tendon or MTJ measures can be considered for routine clinical use, several requirements must be met. Acquisition protocols need to be standardized across devices and operators; age-, sex-, and anatomical-site-specific reference ranges need to be established; and prospective studies must determine whether these measurements predict falls, disability, mobility decline, or treatment responsiveness beyond existing measures of muscle strength, muscle mass, and physical performance. Feasibility, reproducibility, cost, and added clinical value must also be demonstrated in community and primary-care populations. Until such evidence is available, assessment of tendon mechanics and MTJ behavior should be viewed as a complementary research and phenotyping strategy rather than an extension of the formal diagnostic criteria for sarcopenia. In interventional studies, these measurements may also help determine whether improvements in muscle strength are accompanied by changes in tendon mechanics or MTJ behavior, thereby providing a means to evaluate the added value of tissue-informed loading strategies.

## 7 Current limitations, unresolved questions, and future directions

Despite increasing recognition that muscle, tendon, and their interface function as an integrated unit, current research remains largely organized along tissue-specific lines. Most experimental and clinical studies continue to evaluate muscle and tendon independently, with limited attention to their mechanical integration and direct inter-tissue signaling. As a result, an important component of functional decline in sarcopenia remains mechanistically unresolved. An equally important translational limitation is that much of the available evidence for tendon and MTJ remodeling comes from physiological aging, unloading, animal, or *in vitro* studies rather than from cohorts with clinically defined sarcopenia. Consequently, it remains uncertain whether these alterations represent sarcopenia-associated phenotypes or more general consequences of aging and altered mechanical loading. A major limitation is the lack of methodologies that directly quantify muscle-tendon interaction *in vivo*. Although advances in imaging, biomechanics, and spatial omics have begun to provide insight into tissue-specific properties, approaches capable of resolving interface-level function, remodeling dynamics, and force-transfer efficiency remain underdeveloped. This gap constrains both mechanistic understanding and the ability to evaluate therapeutic efficacy at the level of coordinated function.

Several unresolved questions therefore remain. It is still unclear to what extent age-related changes in muscle and tendon reflect true unit-level dysfunction rather than parallel responses to shared systemic drivers. This distinction is further complicated by habitual physical activity and loading history, sex, metabolic disease, and other comorbidities, each of which may independently influence muscle capacity, tendon mechanical properties, and their adaptation to aging. These factors should therefore be considered explicitly when attributing muscle–tendon alterations to sarcopenia itself. Likewise, the precise functional significance of myotendinous junction remodeling in aging has not been fully established, particularly with respect to whether such changes are adaptive, maladaptive, or context dependent. Moreover, the field still lacks robust *in vivo* evidence defining which signaling pathways mediate biologically meaningful muscle-to-tendon and tendon-to-muscle communication during sarcopenia. This limitation is particularly pronounced for tendon-to-muscle regulation, for which current evidence is derived predominantly from co-culture, conditioned-medium, and engineered-interface systems. Future studies should therefore identify tendon-derived mediators and establish their *in vivo* transfer and causal effects on muscle independently of altered mechanical loading.

Resolving the temporal relationship between muscle and tendon degeneration will require study designs that move beyond cross-sectional comparisons of young and old tissues. Longitudinal human studies should preferentially include age-matched individuals with and without clinically defined sarcopenia and combine repeated measurements of muscle strength, muscle quantity, habitual physical activity, loading history, tendon stiffness and elongation, MTJ displacement, and physical performance while accounting for sex, metabolic disease, and relevant comorbidities. Such designs would help determine whether changes in one compartment precede or predict decline in another and, critically, whether tendon or MTJ alterations are specifically associated with sarcopenia rather than reflecting aging, reduced physical activity, or altered loading more generally. Recent advances in ultrasound-based analysis now make several components of this approach increasingly feasible. Automated ultrasound methods can identify and track MTJ displacement during contraction, substantially reducing the dependence on manual frame-by-frame analysis (128, 132). Contemporary studies have also examined the relationship between shear-wave measurements and mechanically derived Achilles tendon stiffness, helping to define both the potential and the methodological limitations of elastography-based tendon assessment (131). These developments provide a practical basis for repeated, non-invasive characterization of muscle-tendon behavior in older populations, although their prognostic value and responsiveness in sarcopenia remain to be established. Experimental studies should further distinguish intrinsic aging from loading-dependent adaptation through controlled unloading and reloading paradigms and tissue-specific manipulation of muscle or tendon, whereas human studies should determine whether these experimentally defined patterns are recapitulated specifically in sarcopenia.

Beyond resolving temporal causality, future research should therefore move toward integrated frameworks that combine mechanical, biological, and spatial resolution. This includes the development of interface-resolved imaging and biomechanical measurements, the application of spatially resolved transcriptomic and proteomic approaches to the myotendinous region, and experimental models that allow controlled manipulation of muscle–tendon coupling. A critical translational priority is to determine whether a muscle–tendon-informed intervention provides benefits beyond conventional resistance exercise. Future trials should compare standard muscle-strengthening programs with loading strategies adjusted according to tendon strain, contraction duration, progression rate, and tissue-specific recovery. Muscle strength, tendon mechanical properties, MTJ behavior, and physical performance should be assessed concurrently to determine whether coordinated adaptation across the unit improves the translation of strength gains into mobility. Until such comparative evidence is available, the principal therapeutic value of the muscle–tendon unit framework lies in refining exercise prescription, patient stratification, and outcome assessment rather than in providing an established alternative to conventional exercise therapy.

More fundamentally, advancing the field will require a shift in conceptual focus. In addition to established muscle-centered determinants, the functional expression of sarcopenia may be influenced by the efficiency with which force is transmitted and coordinated across the muscle–tendon unit. Addressing this gap may be essential for translating structural and molecular insights into meaningful improvements in physical function.

The muscle–tendon unit framework proposed in this Review is not intended to replace the established clinical diagnostic criteria for sarcopenia, which remain appropriately centered on muscle strength, muscle quantity or quality, and physical performance. Instead, complementary assessment of tendon mechanics and MTJ behavior may improve the functional phenotyping of selected populations. Future longitudinal studies should determine whether these measures provide reproducible and clinically meaningful information for patient stratification, prognosis, or treatment monitoring beyond current diagnostic criteria.

## 8 Conclusions

Sarcopenia should be understood not only as a decline in muscle capacity, but also as an impaired translation of that capacity into movement. The muscle–tendon unit framework emphasizes that disrupted coordination between force generation, force transmission, and interface adaptation may contribute to the dissociation between muscle mass, strength, and physical performance. By positioning muscle, tendon, and the MTJ within a continuous functional pathway, this framework links muscle capacity more directly to its functional expression. Rather than redefining sarcopenia, it identifies impaired mechanical and biological integration across the muscle–tendon unit as a testable contributor to functional heterogeneity and treatment response, with potential implications for more precise functional assessment and intervention design.
